# Supplementary material for: Pollen sterols are highly diverse but phylogenetically conserved
Source: New Phytol. 2025 Aug 29;248(2):936–52. doi: 10.1111/nph.70470 (PMC12445805; doi:10.1111/nph.70470)
Supplement: Supplementary file 1 — Fig. S1 Sterol compositions of the species with the highest (left) and lowest (right) Simpson's diversity index as shown in Table S8. Fig. S2 Total sterol production ranged greatly within species sample; graph shows the range in total sterol content across families containing at least three genera. Fig. S3 Median percentage and interquartile range of the most dominant 18 sterol components within each taxonomic unit, ranked by proportion in the sample. Fig. S4 Labelled phylogeny shown in Fig. 2 of main text, covering 261 species adapted from (Zanne et al., 2014) and families with three or more species are highlighted in a clockwise direction. Table S1 Collection details for all pollen samples: multi‐site samples show where multiple under‐weight samples were combined to reach a minimum analysis weight of 10 mg. Table S2 Retention time, mass to charge ratio and structure description of sterols: double bond equivalents used in Quality Assurance mixture. Table S3 Summary of carbon number and double bond positions of sterols identified using reference materials (full structure and formulae for each sterol, except ergosterol, is shown in Fig. 1). Table S4 Species with the highest and lowest total sterol content calculated as species means. Table S5 Mean, SD, maximum and minimum total sterol production of species collected from at least three counties. Table S6 Maximum, minimum, median and interquartile range percentage of sterols which occurred at > 40% in at least one species. Table S7 Maximum, minimum, median and interquartile range of sterols which were present in all taxonomic units analysed. Table S8 Highest and lowest Simpson's diversity index values across all pollen species. Highest values are shown on the left side of the table and lowest values are on the right. Table S9 Sterols most commonly dominant in pollen, defined as occurring within the top 3 sterols by proportion. Table S10 Summary of indicator species analysis (ISA) results comparing the sterol profiles [file NPH-248-936-s001.pdf]

New Phytologist Supporting Information

Article title: Pollen sterols are highly diverse but phylogenetically conserved.

Authors: Ellen C. Baker, Ellen Lamborn, Julia Quiñonez, Stefania Karlsdottir, Abigail Sheppard, Elynor Moore, William E. Kunin, Geraldine A. Wright and Philip C. Stevenson.

NPH-MS-2025-50882

Article acceptance date: 22 July 2025

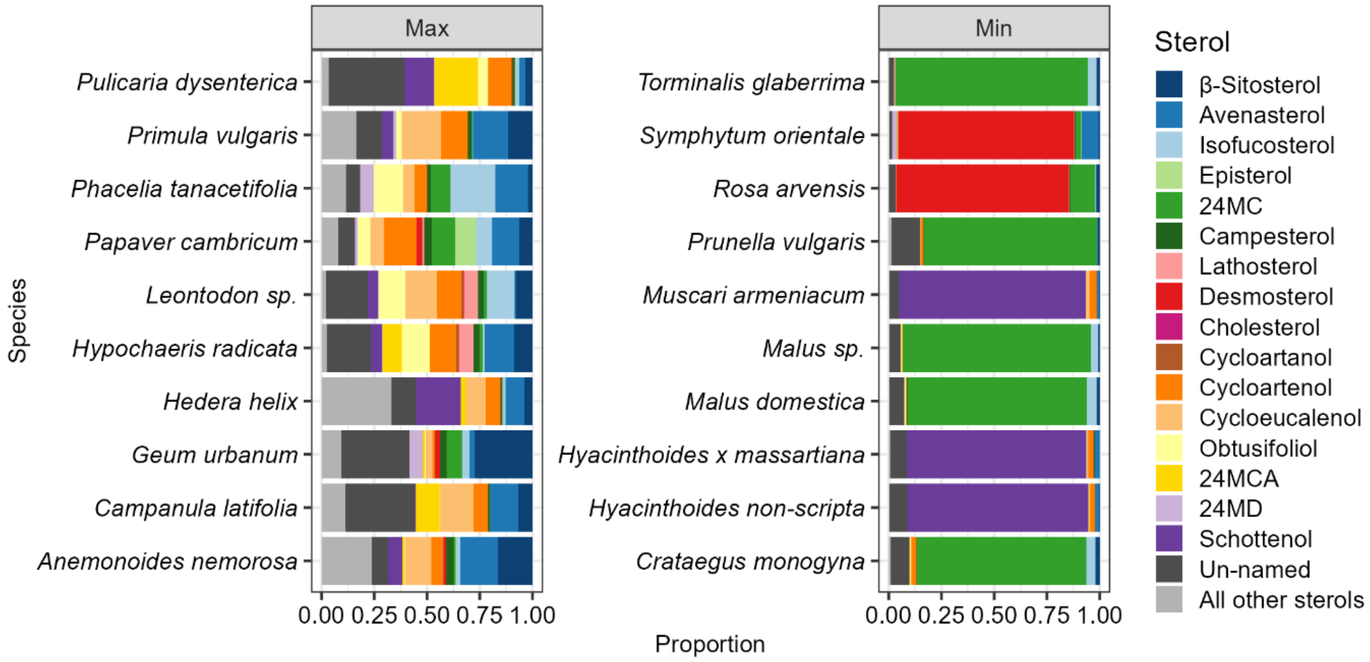

Figure S1. Sterol compositions of the species with the highest (left) and lowest (right) Simpson's diversity index as shown in Table S8. Species with a low diversity index are generally dominated by a single sterol, compared to species with a higher diversity index which produce smaller proportions of a wider range of sterols. Species are arranged alphabetically and sterol colours can be read left to right following legend bottom to top.

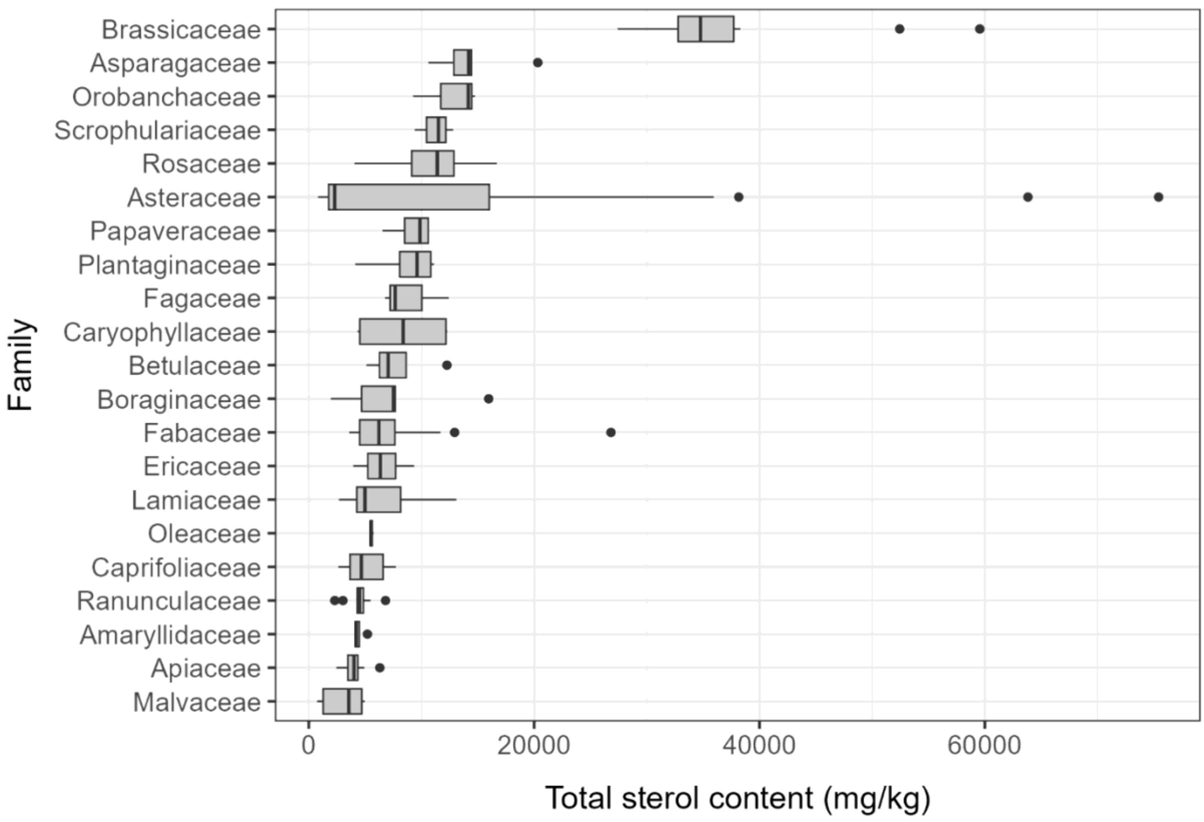

Figure S2. Total sterol production (mg/kg) ranged greatly within species samples. Graph shows the range in total sterol content across families containing at least three genera. Asteraceae is the most sampled family and also displays the highest range in sterol quantity. Brassicaceae shows a less variable high total sterol production. Boxes show first and third quartile, median and 1.5 x inter quartile range. Data is averaged by species, grouped by family and ranked by mean

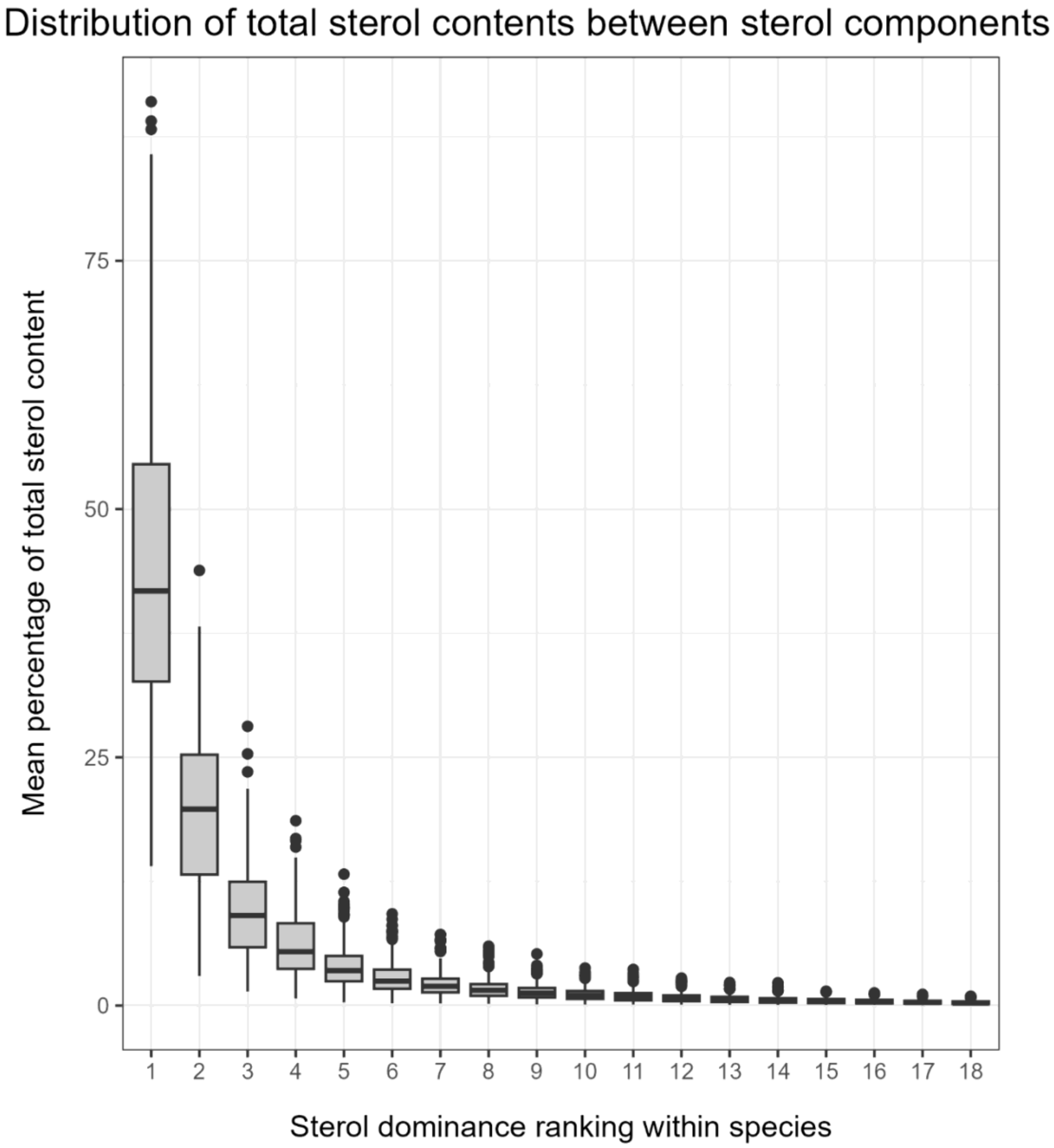

Figure S3. Median percentage and Inter Quartile Range (IQR) of the most dominant 18 sterol components within each taxonomic unit (295), ranked by proportion in the sample. For all taxonomic units, despite most containing >60 sterols, the most dominant 18 sterols accounted for at least 90% of total sterol content. The identity of these 18 sterols varied among taxonomic units.

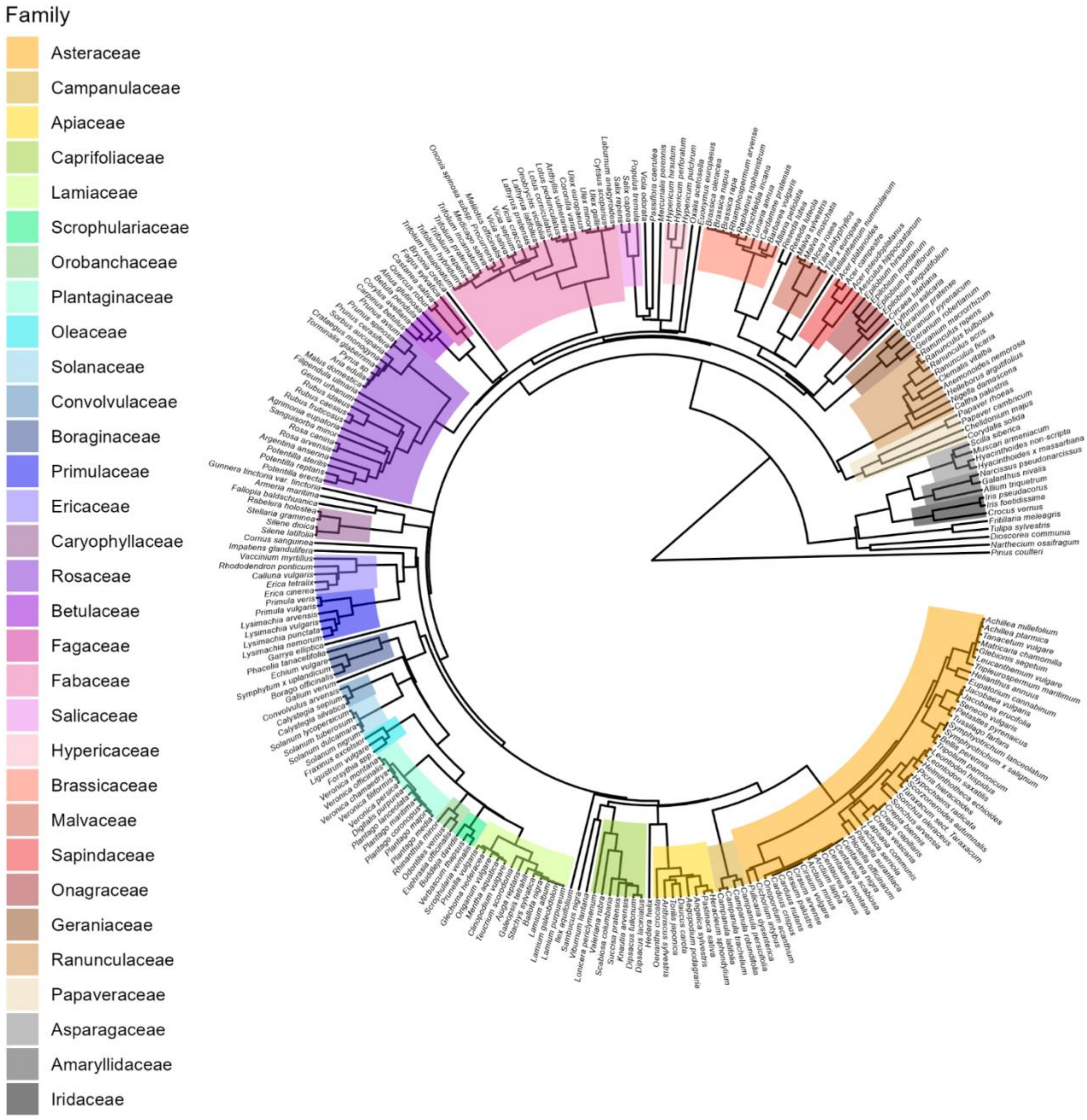

Figure S4. Labelled phylogeny shown in Figure 1 of main text, covering 261 species adapted from (Zanne *et al*., 2014) and families with three or more species are highlighted in a clockwise direction.

Table S1. Collection details for all pollen samples. Multi-site samples show where multiple under-weight samples were combined to reach minimum analysis weight of 10 mg. In these cases, collection month and year are given for each county which formed part of the composite sample. Samples were extracted by collected from 15 counties across February 2021 to September 2022.

Species	County collected	Month collected	Multi-site sample	Samples
Acer campestre L.	Oxfordshire	Apr-22	No	1
Acer platanoides L.	Oxfordshire	Apr-21	No	1
Acer platanoides L.	Sussex	Mar-22	No	1
Acer pseudoplatanus L.	County Durham	Apr-22	No	1
Acer pseudoplatanus L.	County Durham	May-22	No	2
Acer pseudoplatanus L.	Greater London	Apr-22	No	2
Acer pseudoplatanus L.	Oxfordshire	May-21	No	2
Acer sp.	Oxfordshire	Apr-21	No	1
Achillea millefolium L.	Oxfordshire	Aug-21	Yes	1
Achillea millefolium L.	Oxfordshire	Jul-22	Yes	1
Achillea ptarmica L.	Greater London	Jun-22	No	1
Aegopodium podagraria L.	Greater London	May-22	No	1
Aegopodium podagraria L.	Oxfordshire	Jun-22	No	1
Aegopodium podagraria L.	Oxfordshire	May-22	No	1
Aesculus hippocastanum L.	County Durham	May-22	No	1
Aesculus hippocastanum L.	North Yorkshire	May-22	No	1
Aesculus hippocastanum L.	Oxfordshire	May-21	No	2
Agrimonia eupatoria L.	Oxfordshire & Sussex	Jul & Aug - 2021 (O), Jul - 2022 (S)	Yes	1
Ajuga reptans L.	Oxfordshire	May-21	No	1
Alcea rosea L.	Oxfordshire	Aug-21	No	2
Alliaria petiolata (M.Bieb.) Cavara & Grande	Oxfordshire	Apr-22	No	1
Alliaria petiolata (M.Bieb.) Cavara & Grande	Oxfordshire	May-21	No	1
Allium triquetrum L.	Oxfordshire	May-21	No	1
Allium ursinum L.	North Yorkshire	May-22	No	1
Allium ursinum L.	Oxfordshire	Apr-21	No	1
Allium ursinum L.	Oxfordshire	May-21	No	1
Alnus glutinosa (L.) Gaertn.	Greater London	Feb-22	No	3
Alnus glutinosa	Oxfordshire	Feb-22	No	1
Alnus glutinosa	Oxfordshire	Mar-21	No	5
Alnus glutinosa	Oxfordshire	Mar-22	No	1
Anemonoides nemorosa (L.) Holub	Greater London	Mar-22	No	1
Anemonoides nemorosa	Oxfordshire	Apr-22	No	1
Anemonoides nemorosa	Oxfordshire	Mar-22	Yes	1
Anemonoides nemorosa	Sussex	Mar-22	No	1

Anemonoides spp.	West Yorkshire	Apr-21	No	1
Angelica sylvestris L.	Oxfordshire	Aug-21	No	1
Anthriscus sylvestris (L.) Hoffm.	Oxfordshire	Apr-22	No	1
Anthyllis vulneraria L.	County Durham	Jun-22	No	1
Anthyllis vulneraria L.	Sussex	Jul-22	No	1
Arctium agg.	North Yorkshire	Jul-22	No	1
Arctium lappa L.	Oxfordshire	Aug-21	No	2
Arctium lappa L.	Oxfordshire	Jul-22	No	1
Arctium minus (Hill) Bernh.	County Durham	Jul-22	No	1
Arctium minus	Oxfordshire	Aug-21	No	1
Arctium minus	Oxfordshire	Jul-22	No	1
Arctium minus	Sussex	Jul-22	No	1
Argentina anserina (L.) Rydb.	Oxfordshire	May-21	No	1
Aria edulis (Willd.) M.Roem.	Oxfordshire	May-21	No	1
Armeria maritima (Mill.) Willd.	Dorset	Jul-22	No	1
Ballota nigra L.	Oxfordshire	Jul-21	No	1
Ballota nigra L.	Oxfordshire	Sep-21	No	1
Barbarea vulgaris W. T. Aiton	Oxfordshire	May-21	No	1
Bellis perennis L.	Oxfordshire	Feb-21	No	1
Bellis perennis L.	Oxfordshire	Mar-21	No	2
Betula pendula Roth	County Durham	Apr-22	No	2
Betula pendula Roth	North Yorkshire	May-22	No	1
Betula pendula Roth	Oxfordshire	Apr-21	No	2
Borago officinalis L.	Oxfordshire	Aug-21	No	3
Borago officinalis L.	Oxfordshire	Sep-22	No	2
Brassica napus L.	North Yorkshire	Apr-22	No	1
Brassica napus L.	Oxfordshire	Apr-22	No	1
Brassica napus L.	West Yorkshire	Jul-22	No	1
Brassica oleracea L.	Oxfordshire	May-21	No	1
Brassica rapa L.	Oxfordshire	May-22	No	1
Brassica sp.	Oxfordshire	Jun-21	No	1
Brassica sp.	Oxfordshire	May-21	No	1
Bryonia cretica	Oxfordshire	Jun-21	No	1
Bryonia cretica subsp. Dioica	Greater London	Jul-22	No	1
Buddleja davidii Franch.	Oxfordshire	Aug-21	No	1
Buddleja davidii	Oxfordshire	Sep-21	No	1
Calluna vulgaris (L.) Hull	Berkshire	Aug-22	No	1
Calluna vulgaris	Dorset	Jul-22	No	1
Calluna vulgaris	North Yorkshire	Aug-22	No	1
Calluna vulgaris	North Yorkshire	Jul-22	No	1
Calluna vulgaris	Oxfordshire	Sep-21	No	1
Calluna vulgaris	Shropshire	Aug-21	No	1
Caltha palustris L.	Oxfordshire	Apr-21	No	1
Calystegia sepium (L.) R.Br.	Greater London	Jun-22	No	1
Calystegia sepium	North Yorkshire	Jul-22	No	1
Calystegia sepium/sylvatica	Oxfordshire	Aug-21	No	1

Calystegia sepium/sylvatica	Oxfordshire	Sep-21	No	1
Calystegia sylvatica (Kit.) Griseb.	Oxfordshire	Sep-21	No	2
Calystegia sp.	West Yorkshire	Jul-22	No	1
Campanula latifolia L.	North Yorkshire	Jul-22	No	1
Campanula persicifolia L.	Oxfordshire	Jun-21	No	1
Campanula rotundifolia L.	Greater London	Jul-22	No	1
Campanula rotundifolia L.	North Yorkshire	Jul-22	No	2
Campanula trachelium L.	Oxfordshire	Aug-21	No	1
Cardamine pratensis L.	Greater London	Mar-22	No	1
Cardamine pratensis L.	Oxfordshire	Apr-21	No	1
Carduus crispus L.	Oxfordshire	Jul-21	No	1
Carduus nutans L.	Oxfordshire	Jun-21	No	2
Carpinus betulus L.	Greater London	Mar-22	No	1
Carpinus betulus L.	Oxfordshire	Apr-21	No	4
Carpinus betulus L.	Oxfordshire	Mar-21	No	1
Castanea sativa Mill	Greater London	Jul-22	No	1
Centaurea cyanus L.	Dorset	Jul-21	No	1
Centaurea montana L.	Oxfordshire	Jun-21	No	1
Centaurea nigra L.	County Durham	Jul-22	No	2
Centaurea nigra L.	Oxfordshire	Jul-21	No	2
Centaurea nigra L.	Oxfordshire	Jun-21	No	2
Centaurea scabiosa L.	North Yorkshire	Jul-22	No	1
Centaurea scabiosa L.	Oxfordshire	Jul-21	No	1
Centaurea scabiosa L.	Wiltshire	Jul-21	No	1
Chelidonium majus L.	Oxfordshire	May-21	No	1
Cichorium intybus L.	Oxfordshire	Jul-21	No	1
Cichorium intybus L.	Oxfordshire	Jul-21	Yes	1
Circaea lutetiana L.	Oxfordshire	Jul - 2022 & Aug - 2022	Yes	1
Circaea lutetiana L.	Oxfordshire & Greater London	Jul - 2021 (O), Jun - 2022 (GL)	Yes	1
Cirsium acaule (L.) A.A.Weber ex Wigg.	Sussex	Jul-22	No	2
Cirsium arvense (L.) Scop.	County Durham	Jul-22	No	1
Cirsium arvense	Oxfordshire	Jul-22	No	1
Cirsium arvense	Oxfordshire	Jun-21	No	1
Cirsium eriophorum (L.) Scop.	Oxfordshire	Aug-21	No	1
Cirsium eriophorum	Oxfordshire	Aug-22	No	1
Cirsium eriophorum	Wiltshire	Jul-21	No	1
Cirsium palustre (L.) Scop.	North Yorkshire	Aug-22	No	1
Cirsium palustre	North Yorkshire	Jul-22	No	1
Cirsium palustre	Oxfordshire	Jul-21	No	1
Cirsium palustre	Oxfordshire	Jul-22	No	1
Cirsium vulgare (Savi) Ten.	County Durham	Jul-22	No	1
Cirsium vulgare (Savi) Ten.	Greater London	May-22	No	1
Cirsium vulgare	Oxfordshire	Jul-21	No	1

Clematis vitalba L.	Essex	Jun-22	No	1
Clematis vitalba L.	Oxfordshire	Jul-21	No	1
Clinopodium vulgare L.	Oxfordshire	Aug-21	No	1
Convolvulus arvensis L.	Greater London	Jun-22	No	1
Convolvulus arvensis L.	North Yorkshire	Jul-22	No	1
Cornus sanguinea L.	Greater London	May-22	No	1
Cornus sanguinea L.	North Yorkshire	Jun-22	No	1
Cornus sanguinea L.	Oxfordshire	May-22	No	1
Securigera (syn. *Coronilla*) *varia* (L.) Lassen	Oxfordshire	Sep-21	No	1
Corydalis solida (L.) Clairv.	Oxfordshire	Mar-21	No	2
Corylus avellana L.	Greater London	Feb-22	No	1
Corylus avellana L.	Greater London	Jan-22	No	4
Corylus avellana L.	Oxfordshire	Feb-22	No	3
Corylus avellana L.	Oxfordshire	Mar-22	No	1
Crataegus monogyna Jacq.	Greater London	May-22	No	1
Crataegus monogyna Jacq.	Oxfordshire	May-21	No	2
Crepis biennis L.	Oxfordshire	May-21	No	1
Crepis capillaris (L.) Wallr.	County Durham	Jun-22	No	1
Crepis capillaris	Oxfordshire	Jun-22	No	1
Crepis capillaris	Sussex	Jun-22	No	1
Crepis vesicaria L.	North Yorkshire	May-22	No	1
Crepis vesicaria L.	Oxfordshire	Jun-21	No	1
Crocus sp.	Oxfordshire	Mar-21	No	1
Crocus vernus (L.) Hill	Oxfordshire	Feb-21	No	2
Crocus vernus	Oxfordshire	Mar-22	No	2
Crocus x luteus	Oxfordshire	Feb-21	No	1
Cytisus scoparius (L.) Link	County Durham	Apr-22	No	1
Cytisus scoparius	North Yorkshire	May-22	No	1
Cytisus scoparius	Tyne and Wear	Apr-22	No	1
Daucus carota L.	Oxfordshire & North Yorkshire	Jul - 2021(O) , Jul - 2022 (NY)	Yes	1
Digitalis purpurea L.	County Durham	Jun-22	No	1
Digitalis purpurea L.	Oxfordshire	Jul-21	No	1
Dioscorea communis (L.) Caddick & Wilkin	Oxfordshire	Jun-22	No	1
Dipsacus fullonum L.	County Durham	Jul-22	No	1
Dipsacus fullonum L.	County Durham/ North Yorkshire	Aug-22	No	1
Dipsacus fullonum L.	Oxfordshire	Sep-21	No	1
Dipsacus laciniatus L.	Oxfordshire	Aug-21	No	1
Echium vulgare L.	County Durham	Jun-22	No	1
Echium vulgare L.	Oxfordshire	Jul-21	No	1
Echium vulgare L.	Oxfordshire	Jun-21	No	1
Epilobium angustifolium (L.) Scop.	County Durham	Jul-22	No	1
Epilobium angustifolium	Greater London	Jul-22	No	1
Epilobium angustifolium	Oxfordshire	Aug-21	No	1

Epilobium angustifolium	Wiltshire	Jul-21	No	1
Epilobium hirsutum L.	Greater London	Aug-22	No	1
Epilobium hirsutum L.	West Yorkshire	Jul-22	No	1
Epilobium montanum L.	Greater London	May-22	No	1
Epilobium parviflorum Schreb.	West Yorkshire & Sussex	Jun - 2022 (S), Jul - 2022 (WY)	Yes	1
Erica cinerea L.	Berkshire	Aug-22	No	1
Erica cinerea L.	Dorset	Jul-22	No	1
Erica tetralix L.	Berkshire	Aug-22	No	1
Erica tetralix L.	Dorset	Jul-22	No	1
Erica tetralix L.	North Yorkshire	Aug-22	No	1
Euonymus europaeus L.	Oxfordshire	Apr & May - 2022	No	1
Eupatorium cannabinum L.	Oxfordshire	Aug-21	No	2
Euphrasia officinalis L.	County Durham	Aug-22	No	1
Fagus sylvatica L.	Oxfordshire	Apr-21	No	2
Fallopia baldschuanica (Regel) Holub	Oxfordshire	Jul-22	No	1
Filipendula ulmaria (L.) Maxim.	Greater London	Jul-22	No	1
Filipendula ulmaria	Oxfordshire	Jul-21	No	1
Filipendula ulmaria	Sussex	Jun-22	No	1
Filipendula ulmaria	Tyne and Wear	Jun-22	No	1
Forsythia spp.	West Yorkshire	Apr-21	No	1
Fraxinus excelsior L.	Greater London	Apr-22	No	1
Fraxinus excelsior L.	Oxfordshire	Apr-22	No	1
Fritillaria meleagris L.	Oxfordshire	Apr-21	No	2
Galanthus nivalis L.	Oxfordshire	Feb-21	No	2
Galanthus nivalis L.	Oxfordshire	Feb-22	No	1
Galanthus nivalis L.	Oxfordshire	Mar-21	No	2
Galanthus nivalis L.	Oxfordshire	Mar-22	No	1
Galeopsis tetrahit L.	Sussex	Aug-22	No	1
Galium verum	Oxfordshire	Aug-22	No	1
Garrya elliptica Dougl. ex Lindl.	Greater London	Jan-22	No	1
Geranium macrorrhizum L.	Oxfordshire	May-21	No	1
Geranium pratense L.	Oxfordshire	Jun-21	No	1
Geranium pyrenaicum Burm.f.	Oxfordshire	May-21	Yes	1
Geranium robertianum L.	Greater London	Apr-22	No	1
Geranium robertianum L.	Oxfordshire	May-22	No	1
Geum urbanum L.	County Durham & Greater London	May-22	Yes	1
Geum urbanum L.	Oxfordshire	May-22	No	1
Glebionis segetum (L.) Fourr.	Dorset	Jul-21	No	1
Glechoma hederacea L.	County Durham	Apr-22	No	1
Glechoma hederacea L.	Oxfordshire	Apr-21	No	1
Glechoma hederacea	Oxfordshire	May-21	No	1
Gunnera tinctoria var. tinctoria (Molina) Mirb.	Greater London	NA	No	1

Hedera helix	Wiltshire, Oxfordshire & Greater London	Sep - 2021 (W & O), Sep - 2022 (GL)	Yes	1
Helianthemum nummularium (L.) Mill.	Oxfordshire	Jun-21	Yes	1
Helianthemum nummularium (L.) Mill.	Oxfordshire	Sep-21	No	1
Helianthus annuus L.	NA	Sep-21	No	4
Helleborus argutifolius Viv.	Oxfordshire	Apr-21	No	1
Helminthotheca echioides (L.) Holub	Oxfordshire	Jul-21	No	1
Helminthotheca echioides (L.) Holub	Oxfordshire	Sep-22	No	1
Heracleum sphondylium L.	Greater London	Jun-22	No	1
Heracleum sphondylium L.	Oxfordshire	Jun-21	No	1
Hirschfeldia incana (L.) Lagr.-Foss.	North Yorkshire	Jun-22	No	1
Hyacinthoides non-scripta (L) Chouard *ex* Rothm.	County Durham	Apr-22	No	1
Hyacinthoides non-scripta	Greater London	Apr-22	No	1
Hyacinthoides non-scripta	North Yorkshire	Apr-22	No	1
Hyacinthoides non-scripta	North Yorkshire	May-22	No	1
Hyacinthoides non-scripta	Oxfordshire	Apr-21	No	2
Hyacinthoides non-scripta	Oxfordshire	Apr-22	No	1
Hyacinthoides non-scripta	Oxfordshire	May-21	No	2
Hyacinthoides x massartiana	County Durham	Apr-22	No	1
Hyacinthoides x massartiana	North Yorkshire	May-22	No	1
Hyacinthoides x massartiana	Oxfordshire	Apr-21	No	1
Hypericum hirsutum L.	Oxfordshire	Jul-21	Yes	1
Hypericum perforatum L.	North Yorkshire	Aug & Sep - 2022	No	1
Hypericum perforatum L.	Oxfordshire	Jul-21	No	1
Hypericum pulchrum L.	Sussex	Jun-22	No	1
Hypochaeris radicata L.	North Yorkshire	Jun-22	No	1
Hypochaeris radicata L.	Oxfordshire	Jun-21	No	1
Hypochaeris radicata L.	Oxfordshire	Jun-22	No	1
Hypochaeris radicata L.	Oxfordshire & Greater London	Jun - 2021 (O), Jul - 2022 (GL)	Yes	1
Ilex aquifolium L.	Greater London	Apr-22	No	1
Ilex aquifolium	Oxfordshire	May-22	No	1
Impatiens glandulifera Royle	County Durham	Sep-22	No	1
Impatiens glandulifera	Greater London	Aug-22	No	1
Impatiens glandulifera	Oxfordshire	Jul-21	No	1
Iris foetidissima L.	Oxfordshire	Jun-22	No	1
Iris pseudacorus L.	Greater London	May-22	No	2
Iris pseudacorus L.	Oxfordshire	May-22	No	1
Jacobaea erucifolia (L) G.Gaertn., B.Mey. & Scherb.	County Durham	Aug-22	No	1
Jacobaea erucifolia	Greater London	Jul-22	No	1
Jacobaea erucifolia	Oxfordshire	Aug-22	No	1

Jacobaea erucifolia	Wiltshire	Sep-21	No	1
Jacobaea vulgaris Gaertn.	Greater London	Jun-22	No	1
Jacobaea vulgaris	North Yorkshire	Jul-22	No	1
Jacobaea vulgaris	Oxfordshire	Jun-22	No	1
Jacobaea vulgaris	Sussex	Jul-22	No	1
Knautia arvensis (L) Coult.	County Durham	Jul-22	No	1
Knautia arvensis	North Yorkshire	Jul-22	No	1
Knautia arvensis	Oxfordshire	Jul-21	No	2
Knautia arvensis	Oxfordshire	Jun-21	No	1
Knautia arvensis	Wiltshire	Jul-21	No	1
Laburnum anagyroides Medik.,	Oxfordshire	May-21	No	1
Lactuca serriola L.	Oxfordshire	Sep-21	No	1
Lamium album L.	County Durham	Apr-22	No	1
Lamium album L.	Oxfordshire	Apr-21	No	1
Lamium album L.	Oxfordshire	Aug-21	No	2
Lamium galeobdolon L.	Oxfordshire	Apr-21	No	1
Lamium galeobdolon subsp. Argentatum	Oxfordshire	Apr-22	No	1
Lamium purpureum L.	Greater London	Apr-22	No	1
Lapsana communis L.	County Durham	Jun-22	No	1
Lapsana communis L.	Oxfordshire	Jun-21	Yes	1
Lapsana communis L.	Oxfordshire	Sep-21	Yes	1
Lathyrus latifolius L.	Oxfordshire	Jul-21	No	2
Lathyrus pratensis L.	Oxfordshire	Jul-21	Yes	1
Lathyrus pratensis L.	Sussex	Jun-22	No	1
Leontodon hispidus L.	Oxfordshire	Jun-22	No	1
Leontodon hispidus L.	Oxfordshire	Sep-21	No	1
Leontodon hispidus L.	Sussex	Jun-22	No	1
Leontodon saxatilis Lam.	Greater London	Jun-22	No	1
Leontodon sp.	Oxfordshire	Aug-21	No	1
Leucanthemum vulgare Lam.	North Yorkshire	May-22	No	1
Leucanthemum vulgare Lam.	Oxfordshire	Jul-21	No	1
Leucanthemum vulgare Lam.	Oxfordshire	Jun-21	No	2
Leucanthemum vulgare Lam.	Oxfordshire	May & Jun - 2021	Yes	1
Leucanthemum vulgare Lam.	Sussex	Jun-22	No	1
Ligustrum sp.	Oxfordshire	Jul-21	No	1
Ligustrum vulgare L.	Oxfordshire	Jun-22	No	1
Lonicera caprifolium L.	Oxfordshire	Jun-21	No	1
Lonicera periclymenum L.	North Yorkshire	Aug-22	No	1
Lonicera periclymenum L.	Oxfordshire	Jun-21	No	1
Lotus corniculatus L.	North Yorkshire	Jun-22	No	1
Lotus corniculatus L.	Oxfordshire	Aug-21	No	1
Lotus corniculatus L.	Oxfordshire	Jul & Aug - 2021	Yes	1
Lotus corniculatus L.	Oxfordshire	Jun-21	No	1
Lotus corniculatus L.	Oxfordshire	Jun-21	Yes	1
Lotus corniculatus L.	Oxfordshire	Sep-22	No	2

Lotus corniculatus subsp. corniculatus L.	North Yorkshire	May-22	No	1
Lotus pedunculatus Cav.	North Yorkshire	Jul-22	No	1
Lotus pedunculatus	Sussex	June & July - 2022	Yes	1
Lotus pedunculatus	Sussex	Jun-22	No	1
Lunaria annua L.	Oxfordshire	May-21	No	1
Lysimachia arvensis (L.) U.Manns & Anderb.	County Durham	Jun-22	No	1
Lysimachia nemorum L.	Oxfordshire & Sussex	Jul - 2022 (O), Jun - 2022 (S)	Yes	1
Lysimachia punctata L.	Oxfordshire	Jul-21	No	1
Lysimachia vulgaris L.	Oxfordshire	Aug-21	No	1
Lythrum salicaria L.	Oxfordshire	Aug & Sep - 2021	Yes	1
Malus domestica L.	County Durham	Apr-22	No	1
Malus domestica (Suckow) Borkh.,	Greater London	Apr-22	No	1
Malus domestica	North Yorkshire	Apr-22	No	1
Malus domestica	Oxfordshire	May-21	No	1
Malus sp.	Oxfordshire	Apr-22	No	3
Malva moschata L.	Dorset	Jul-21	No	1
Malva moschata L.	North Yorkshire	Jul-22	No	1
Malva moschata L.	Oxfordshire	Jul-21	No	2
Malva moschata L.	Oxfordshire	Jun-21	No	1
Malva sylvestris L.	Greater London	Jun-22	No	1
Malva sylvestris L.	Oxfordshire	Jun-21	No	4
Matricaria chamomilla L.	County Durham	May-22	No	1
Matricaria chamomilla L.	Oxfordshire	Aug-21	No	1
Medicago sativa L.	Oxfordshire	Sep-21	No	1
Medicago sativa L.	Oxfordshire	Sep-22	No	1
Melilotus officinalis (L.) Lam.	Oxfordshire	Aug-21	No	1
Melilotus sp.	Oxfordshire	Aug-21	No	2
Mentha aquatica L.	County Durham	Aug-22	No	1
Mentha aquatica L.	Oxfordshire	Aug-21	No	1
Mercurialis perennis L.	Greater London	Apr-22	No	1
Mercurialis perennis L.	North Yorkshire	Apr-22	No	2
Mercurialis perennis L.	Oxfordshire	Apr-21	No	2
Muscari armeniacum Leichtlin	Wiltshire	Apr-21	No	1
Narcissus pseudonarcissus L.	Greater London	Feb-22	No	1
Narcissus sp.	Oxfordshire	Apr-21	No	1
Narcissus sp.	Oxfordshire	Feb-21	No	3
Narcissus sp.	Oxfordshire	Mar-21	No	4
Narcissus sp.	Oxfordshire	Mar-22	No	2
Narthecium ossifragum (L.) Huds.	Northumberland	Jul-22	No	1
Nigella damascena L.	Wiltshire	Sep-21	No	1
Odontites vernus Dumort.	Oxfordshire	Aug-21	No	1
Odontites vernus Dumort.	Wiltshire	Jul-21	No	1

Odontites vernus	Wiltshire & Oxfordshire	Aug - 2021 (W & O)	Yes	1
Oenanthe crocata L.	Greater London	May-22	No	1
Oenanthe crocata L.	Oxfordshire	May-22	No	1
Onobrychis viciifolia Scop.	County Durham	Jul-22	No	1
Onobrychis viciifolia	Wiltshire	Aug-21	No	1
Onobrychis viciifolia	Wiltshire	Jul-21	No	1
Ononis spinosa subsp. procurrens L.	North Yorkshire	Jun-22	No	1
Ononis spinosa subsp. procurrens L.	Oxfordshire	Sep-21	No	1
Onopordum acanthium L.	Oxfordshire	Jul-21	No	1
Origanum vulgare L.	Oxfordshire	Aug-21	No	1
Oxalis acetosella L.	North Yorkshire	May-22	No	1
Papaver cambricum L.	Oxfordshire	Jun-21	No	1
Papaver cambricum L.	Oxfordshire	May-21	No	1
Papaver rhoeas L.	Greater London	Jun-22	No	1
Papaver rhoeas L.	Oxfordshire	Jun-21	No	1
Passiflora caerulea L.	Oxfordshire	Aug-21	No	1
Pastinaca sativa L.	County Durham	Jul-22	No	1
Pastinaca sativa L.	Oxfordshire	Jul-22	No	1
Petasites hybridus L.	North Yorkshire	Apr-22	No	2
Petasites hybridus L.	Oxfordshire	Apr-22	No	2
Petasites pyrenaicus (L.) G.Gaertn., B.Mey. & Scherb.	Oxfordshire	Feb-22	No	1
Phacelia tanacetifolia Benth.	Oxfordshire	Sep-21	No	1
Picris hieracioides L.	Oxfordshire	Aug-21	No	1
Pilosella aurantiaca (L.) F.W.Schultz & Sch.Bip	County Durham	Jun-22	No	1
Pilosella officinarum Vaill.	Oxfordshire	Jun-22	No	1
Pilosella officinarum Vaill.	Oxfordshire, County Durham & Greater London	Aug - 2021 (O), Jun - 2022 (CD), May - 2022 (GL)	Yes	1
Pinus coulteri D. Don.	Greater London	2022	No	1
Plantago coronopus L.	Dorset	Jul-22	No	1
Plantago coronopus L.	Greater London	May-22	No	1
Plantago coronopus L.	North Yorkshire	Jun-22	No	1
Plantago lanceolata L.	Greater London	May-22	No	1
Plantago lanceolata L.	North Yorkshire	May-22	No	2
Plantago lanceolata L.	Oxfordshire	Aug-21	No	1
Plantago lanceolata L.	Oxfordshire	May-21	No	3
Plantago major L.	Oxfordshire	Jul-21	No	1
Plantago maritima L.	County Durham	Jun-22	No	1
Plantago maritima L.	Dorset	Jul-22	No	1
Plantago media L.	Oxfordshire	Jun & Jul - 2021	Yes	1
Plantago media L.	Oxfordshire	Jun-21	No	1
Plantago media L.	Oxfordshire	Jun - 2021, Aug - 2021	Yes	1

Populus tremula L.	Oxfordshire	Mar-22	Yes	1
Potentilla erecta (L.) Raeusch.	Berkshire	May-22	No	1
Potentilla reptans L.	Oxfordshire	Jun-22	No	1
Potentilla sterilis (L.) Garcke	Oxfordshire & North Yorkshire	Apr - 2022 (O), Mar - 2022 (NY)	Yes	1
Primula veris L.	Oxfordshire	Apr-21	No	2
Primula vulgaris Huds.	County Durham & North Yorkshire	Apr - 2022 (CD & NY)	Yes	1
Primula vulgaris Huds.	Oxfordshire	Feb-21	No	1
Primula vulgaris Huds.	Oxfordshire	Mar-21	No	4
Primula vulgaris Huds.	Oxfordshire	Mar-22	No	1
Primula vulgaris Huds.	Sussex	Mar-22	No	1
Prunella vulgaris Huds.	Oxfordshire	Jun-22	No	1
Prunus avium L.	Greater London	Mar-22	No	1
Prunus avium L.	Oxfordshire	Apr-21	No	2
Prunus avium L.	Oxfordshire	Mar-21	No	1
Prunus avium L.	Sussex	Mar-22	No	1
Prunus cerasifera Ehrh.	Oxfordshire	Mar-21	No	6
Prunus cerasifera Ehrh.	Oxfordshire	Mar-22	No	1
Prunus spinosa L.	Berkshire	Apr-21	No	1
Prunus spinosa L.	County Durham	Apr-22	Yes	1
Prunus spinosa L.	Oxfordshire	Apr-21	No	1
Prunus spinosa L.	Oxfordshire	Mar-21	Yes	1
Prunus spinosa L.	Sussex	Mar-22	No	1
Pulicaria dysenterica (L.) Bernh.	Oxfordshire	Aug-21	No	1
Pulicaria dysenterica	Oxfordshire & North Yorkshire	Sep - 2021 (O), Sep - 2022 (NY)	Yes	1
Pulicaria dysenterica	Sussex	Aug-22	No	1
Pulicaria dysenterica	Wiltshire	Sep-21	No	1
Pyrus sp.	Oxfordshire	Apr-21	No	1
Quercus robur L.	Greater London	Apr-22	No	1
Rabelera holostea M.T.Sharples & E.A.Tripp	North Yorkshire	Apr-22	No	1
Rabelera holostea M.T.Sharples & E.A.Tripp	North Yorkshire	May-22	No	1
Rabelera holostea	Oxfordshire	Apr-22	No	1
Rabelera holostea	Oxfordshire	May-21	No	3
Ranunculus acris L.	County Durham	May-22	No	1
Ranunculus acris L.	Greater London	May-22	No	2
Ranunculus acris L.	North Yorkshire	Jun-22	No	1
Ranunculus acris L.	North Yorkshire	May-22	No	1
Ranunculus acris L.	Oxfordshire	Jun-21	No	1
Ranunculus acris L.	Oxfordshire	May-21	No	1
Ranunculus bulbosus L.	Greater London	Apr-22	No	1
Ranunculus bulbosus L.	Greater London	NA	No	1
Ranunculus bulbosus L.	North Yorkshire	May-22	No	1
Ranunculus bulbosus L.	Oxfordshire	Apr-21	No	2
Ranunculus bulbosus L.	Oxfordshire	May-21	No	1

Ranunculus ficaria Huds.	Greater London	Mar-22	No	1
Ranunculus ficaria Huds.	North Yorkshire	Apr-22	No	1
Ranunculus ficaria Huds.	North Yorkshire	Mar-22	No	1
Ranunculus ficaria Huds.	Oxfordshire	Mar-21	No	4
Ranunculus ficaria Huds.	Wiltshire	Mar-22	No	1
Ranunculus repens L.	Greater London	May-22	No	1
Ranunculus repens L.	North Yorkshire	Jun-22	No	1
Ranunculus repens L.	Oxfordshire	Jul-21	No	1
Ranunculus repens L.	Oxfordshire	May-21	No	1
Ranunculus sp.	Oxfordshire	Mar-21	No	1
Raphanus raphanistrum L.	Greater London	Jun-22	No	1
Raphanus raphanistrum subsp. Sativus L.	Greater London	Sep-22	No	1
Reseda lutea L.	County Durham	Jul-22	No	1
Reseda lutea L.	Greater London	May-22	No	1
Reseda lutea L.	North Yorkshire	Jun-22	No	1
Reseda luteola L.	Greater London	May-22	No	1
Reseda luteola L.	Tyne and Wear	May-22	No	1
Rhamphospermum arvense Andrz. ex Besser	North Yorkshire	May-22	No	1
Rhamphospermum arvense	Oxfordshire	Apr-22	No	1
Rhamphospermum arvense	Oxfordshire	Jun-22	No	1
Rhinanthus minor L.	North Yorkshire	Jun-22	No	1
Rhinanthus minor L.	Oxfordshire	Jul-21	No	1
Rhinanthus minor L.	Oxfordshire	Jun-21	No	2
Rhododendron ponticum L.	Greater London	May-22	No	1
Rhododendron ponticum L.	Oxfordshire	Jun-21	No	1
Rosa arvensis Huds.	Sussex	Jun-22	No	2
Rosa canina L.	Greater London	May-22	No	1
Rosa canina L.	North Yorkshire	Jun-22	No	1
Rosa canina L.	Oxfordshire	Jun-21	Yes	1
Rosa canina L.	Oxfordshire	Jun-22	No	1
Rubus caesius L.	Wiltshire	Sep-21	No	1
Rubus fruticosus L.	Oxfordshire	Aug-21	No	1
Rubus fruticosus L.	Oxfordshire	Jun-21	No	1
Rubus fruticosus L.	Wiltshire	Jul-21	No	1
Rubus idaeus L.	Oxfordshire	Jun-22	No	1
Rubus idaeus L.	Sussex	Jun-22	No	1
Salix caprea L.	Oxfordshire	Mar-21	No	1
Salix caprea L.	Oxfordshire	Mar-22	No	1
Salix caprea â€œKilmarnockâ€	Greater London	Mar-22	No	1
Salix caprea/cinerea	Berkshire	Apr-21	No	1
Salix caprea/cinerea	County Durham	Apr-22	No	3
Salix caprea/cinerea	Oxfordshire	Mar-21	No	4
Salix caprea/cinerea	Sussex	Mar-22	No	1
Salix fragilis/alba L.	Greater London	Apr-22	No	1
Salix repens L.	Greater London	Apr-22	No	1

Salix sp.	County Durham	Apr-22	No	1
Salix sp.	Oxfordshire	Apr-22	No	2
Salix sp.	Oxfordshire	Mar-21	No	2
Salix x pendulina nothof. Tristis	Oxfordshire	Apr-21	No	2
Sambucus nigra L.	Oxfordshire	Jun-21	No	3
Sanguisorba minor Scop.	County Durham	Jun-22	No	1
Sanguisorba minor Scop.	Greater London	Jun-22	No	2
Sanguisorba minor Scop.	Oxfordshire	Apr-22	No	1
Scabiosa columbaria L.	Wiltshire	Jul-21	No	1
Scilla forbesii (Baker) Speta	Oxfordshire	Mar-21	No	2
Scilla siberica Andrews	Oxfordshire	Mar-21	No	1
Scorzoneroides autumnalis (L.) Moench	County Durham	Sep-22	No	1
Scorzoneroides autumnalis	Greater London	Jul-22	No	1
Scorzoneroides autumnalis	Oxfordshire	Aug-21	Yes	1
Scrophularia vernalis L.	Oxfordshire	May-21	No	1
Senecio vulgaris L.	Greater London & North Yorkshire	Jun - 2022 (NY), Aug - 2022 (GL)	Yes	1
Senecio/Jacobea sp.	Oxfordshire	Jul-21	No	2
Silene dioica (L.) Clairv.	County Durham	May-22	No	2
Silene dioica	North Yorkshire	May-22	No	1
Silene dioica	Oxfordshire	Jun-21	No	1
Silene dioica	Oxfordshire	Jun, Jul & Aug - 2021	Yes	1
Silene dioica	Oxfordshire	May-21	No	1
Silene latifolia Poir.	Oxfordshire	Aug-21	No	1
Silene latifolia	Oxfordshire	Jun-21	No	1
Solanum dulcamara L.	Greater London	May-22	No	1
Solanum dulcamara L.	Oxfordshire	Jun-22	No	1
Solanum lycopersicum L.	NA	Jul-21	No	1
Solanum nigrum L.	Greater London	Jun-22	No	1
Solanum tuberosum L.	North Yorkshire	Jul-22	No	1
Sonchus arvensis L.	County Durham	Aug-22	No	1
Sonchus arvensis L.	North Yorkshire	Jun-22	No	1
Sonchus arvensis L.	Sussex	Jul-22	No	1
Sonchus oleraceus L.	Greater London	May-22	No	1
Sonchus oleraceus L.	Oxfordshire, County Durham & North Yorkshire	Jun - 2022 (NY), Jul - 2022 (CD), Jul - 2022 (O)	Yes	1
Sorbus aucuparia L.	County Durham	May-22	No	1
Sorbus aucuparia L.	Oxfordshire	May-21	No	3
Stachys sylvatica L.	Oxfordshire	Jun-22	No	1
Stachys sylvatica L.	Sussex	Jun-22	No	1
Stellaria graminea L.	Oxfordshire	Jun-22	No	1
Succisa pratensis Moench	County Durham	Aug-22	No	1
Succisa pratensis	Greater London	Sep-22	No	1
Succisa pratensis	Oxfordshire	Aug-21	No	1
Succisa pratensis	Oxfordshire	Sep-21	No	1

Symphyotrichum lanceolatum (Willd.) G.L.Nesom	Oxfordshire	Sep-21	No	2
Symphyotrichum x salignum	County Durham	Sep-22	No	1
Symphytum orientale L.	Oxfordshire	Apr-21	No	1
Symphytum sp.	Oxfordshire	Jun-21	No	1
Symphytum x uplandicum	Greater London	May-22	No	1
Tanacetum vulgare L.	County Durham	Jul-22	No	1
Tanacetum vulgare L.	County Durham	Sep-22	No	1
Tanacetum vulgare L.	North Yorkshire	Jul-22	No	1
Taraxacum sect. Taraxacum	Greater London	Apr-22	No	1
Taraxacum sect. Taraxacum	Oxfordshire	Apr-21	No	1
Teucrium scorodonia L.	Sussex	Jun-22	No	1
Tilia platyphyllos Scop.	Greater London	Jun-22	No	3
Tilia x europaea	Oxfordshire	Jun-21	No	1
Torilis japonica (Houtt.) DC.	North Yorkshire	Jul-22	No	1
Torminalis glaberrima (Gand.) Sennikov & Kurtto	Greater London	May-22	No	1
Trifolium hybridum L.	Oxfordshire	Sep-22	No	1
Trifolium incarnatum L.	Oxfordshire	Sep-22	No	3
Trifolium pratense L.	Oxfordshire	Jul-21	No	4
Trifolium pratense L.	Oxfordshire	Jul-21	Yes	1
Trifolium pratense L.	Oxfordshire	Sep-21	No	1
Trifolium pratense L.	Oxfordshire	Sep-22	No	3
Trifolium repens L.	Oxfordshire	Aug-21	No	3
Trifolium repens L.	Oxfordshire	Sep-22	No	1
Trifolium resupinatum L.	Oxfordshire	Sep-22	No	2
Tripleurospermum inodorum (L.) Sch.Bip.	Oxfordshire	Jul-21	No	1
Tripleurospermum inodorum	Oxfordshire	Sep-22	No	1
Tripleurospermum maritimum	Dorset	Jul-22	No	1
Tripolium pannonicum (Jacq.) Dobrocz.	County Durham	Aug-22	No	1
Tripolium pannonicum	County Durham/North Yorkshire	Aug-22	No	1
Tripolium pannonicum	Essex	Sep-22	No	2
Tripolium pannonicum	Kent	Sep-22	No	1
Tulipa sylvestris L.	Oxfordshire	Apr-21	No	3
Tussilago farfara L.	County Durham	Mar-22	No	1
Ulex europaeus L.	Greater London	Mar-22	No	1
Ulex europaeus L.	Oxfordshire	Mar-22	No	1
Ulex europaeus L.	Oxfordshire	May-21	No	2
Ulex europaeus L.	Sussex	Mar-22	No	1
Ulex gallii Planch.	Dorset	Sep-22	No	1
Ulex minor L.	Berkshire & Dorset	Aug - 2022 (B), Sep - 2022 (D)	Yes	1
Ulex minor Roth.	Hampshire	Aug-22	No	1
Vaccinium myrtillus L.	Berkshire	May-22	No	1

Vaccinium myrtillus L.	North Yorkshire	May-22	No	2
Valeriana rubra L.	Oxfordshire	Jun-21	No	2
Verbascum thapsus L.	Oxfordshire	Jul - 2021, Aug - 2021	Yes	1
Veronica chamaedrys L.	Greater London	May-22	No	1
Veronica chamaedrys L.	North Yorkshire	May-22	No	1
Veronica chamaedrys L.	Oxfordshire	May-21	No	1
Veronica filiformis Sm.	Oxfordshire	Apr-22	No	1
Veronica montana L.	Oxfordshire	May-22	No	1
Veronica officinalis L.	Oxfordshire	Jun-22	No	1
Veronica officinalis L.	Sussex	Jun-22	No	1
Veronica persica Poir.	Oxfordshire	Mar-21	No	1
Viburnum lantana L.	Oxfordshire	Apr-21	No	1
Viburnum lantana L.	Oxfordshire	May-21	No	1
Vicia cracca L.	Oxfordshire	Jul - 2021, Aug - 2021, Jul - 2022, Aug - 2022	Yes	1
Vicia sativa L.	Oxfordshire	May-22	No	1
Vicia sativa L.	Oxfordshire, County Durham & North Yorkshire	May - 2021 (O), Apr - 2022 (O), Jun - 2022 (NY), May - 2022 (CD)	Yes	1
Vicia sepium L.	North Yorkshire	Apr-22	No	1
Vicia sepium L.	Oxfordshire	May-22	No	2
Viola odorata L.	Oxfordshire	Mar-21	No	1
Viola odorata L.	Oxfordshire	Mar - 2021, Mar - 2022	Yes	1
Viola riviniana Rchb.	North Yorkshire	Apr-22	No	1
Viola riviniana	Sussex	Apr-22	No	1

Table S2. Retention time, mass to charge ratio (m/z) and structure description of sterols (carbon count (CC): double bond equivalents (DB)) used in Quality Assurance (QA) mixture.

Sterol	Structure	m/z	Retention time (min)
24-methylenecholesterol (24MC)	(28:2)	398.3549	5.9
24-methylenecycloartanol (24MCA)	(31:2)	440.4018	7.5
Anthelsterol	(29:3)	410.3549	5.3
Avenasterol	(29:2)	412.3705	6.9
β-sitosterol	(29:1)	414.3862	9.7
Brassicasterol	(28:2)	398.3549	6.6
Campesterol	(28:1)	400.3705	8.2
Cholesterol	(27:1)	386.3549	7.0
Cycloartenol	(30:2)	426.3862	8.3
Cycloeucalenol	(30:2)	426.3862	7.7
Cyclolaudenol	(31:2)	440.4018	9.5
Desmosterol	(27:2)	384.3392	5.1
Episterol	(28:2)	398.3549	5.7
Ergosterol	(28:3)	396.3392	5.5
Isofucosterol	(29:2)	412.3705	7.5
Schottenol	(29:1)	414.3862	9.0
Sitostanol	(29:0)	416.4018	11.1
Spinasterol	(29:2)	412.3705	8.0
Stigmasterol	(29:2)	412.3705	8.5

Table S3. Summary of carbon count double bond positions of sterols identified using reference materials. Full structure and formula for each sterol, except ergosterol, is shown in Figure 2. Ergosterol is classed as NA due to it containing double bonds at positions five and seven in the steroid nucleus B-ring. CPR = Cyclopropane ring.

Sterol	Carbons	Double bond
Cholesterol	27	5
Lathosterol	27	7
Desmosterol	27	5
Campesterol	28	5
Lophenol	28	7
24MC	28	5
24MD	28	5
Brassicasterol	28	5
Episterol	28	7
Ergosterol	28	NA
Sitostanol	29	0
β-Sitosterol	29	5
Schottenol	29	7
24MP	29	CPR
Avenasterol	29	7
Isofucosterol	29	5
Spinasterol	29	7
Stigmasterol	29	5
Cycloartanol	30	CPR
24MCA	30	CPR
Cycloartenol	30	CPR
Cycloeucalenol	30	CPR
Obtusifoliol	30	8
Cyclolaudenol	31	CPR

Table S4. Species with the highest and lowest total sterol content (mg/kg). Values were calculated as species means. Highlighted rows belong to the family Asteraceae and contain some of the highest and lowest values in the dataset. Brassicaceae species also showed consistently high total sterol production (*Lunaria annua*, *Cardamine pratensis*, *Brassica rapa*).

Species	Total Sterol (mg/kg)	Species	Total Sterol (mg/kg)
Taraxacum sect. Taraxacum	75423.52	*Malva sylvestris*	729.98
Lapsana communis	63824.55	*Centaurea cyanus*	827.78
Lunaria annua	59551.78	*Bellis perennis*	831.20
Cardamine pratensis	52441.40	*Carduus nutans*	1072.10
Brassica rapa	38312.12	*Pinus coulteri*	1136.28

Table S5. Mean, standard deviation, maximum and minimum total sterol production (mg/kg) of species collected from at least three counties. Data shows large variation in total sterol production between species. Total sterol production also varied within species despite a largely consistent sterol profile. This intra-species variation was not found to be significantly influenced by collection county.

Species	Total samples	Counties collected from	Mean (mg/kg)	SD (mg/kg)	Max (mg/kg)	Min (mg/kg)
Acer pseudoplatanus	7	3	8610.56	1345.59	10928.20	6953.34
Aesculus hippocastanum	4	3	4604.56	547.78	5235.14	4002.78
Anemonoides nemorosa	4	3	2307.65	170.94	2524.54	2127.58
Arctium minus	4	3	2219.32	704.02	3265.20	1739.24
Betula pendula	5	3	5116.40	2029.07	6597.53	1589.77
Brassica napus	3	3	33451.56	3396.65	37013.05	30248.11
Calluna vulgaris	6	5	6348.89	1154.77	7264.60	4134.19
Centaurea scabiosa	3	3	1199.63	216.62	1449.55	1065.77
Cirsium vulgare	3	3	1709.68	211.23	1953.15	1575.38
Cornus sanguinea	3	3	3930.19	1241.60	5233.46	2761.16
Crepis capillaris	3	3	22428.86	3475.13	25239.07	18543.12
Cytisus scoparius	3	3	4110.61	972.25	5233.26	3544.54
Dipsacus fullonum	3	3	4412.75	449.28	4812.91	3926.74
Epilobium angustifolium	4	4	8474.81	1572.14	10182.08	6475.26
Erica tetralix	3	3	5238.58	1340.62	6386.28	3765.09
Filipendula ulmaria	4	4	8687.12	2123.70	11335.50	6174.78
Hyacinthoides non-scripta	9	4	14194.95	2261.07	16081.94	10057.01
Hyacinthoides x massartiana	3	3	12877.68	202.28	13110.38	12743.78
Hypochaeris radicata	4	3	6191.68	595.63	6762.11	5397.87
Impatiens glandulifera	3	3	2591.73	303.33	2939.30	2380.48
Jacobaea erucifolia	4	4	1870.75	460.03	2323.47	1283.10
Jacobaea vulgaris	4	4	1895.42	325.24	2235.33	1568.93
Knautia arvensis	6	4	3593.10	298.44	4050.03	3207.38
Leucanthemum vulgare	6	3	2171.35	207.51	2517.86	1977.68
Malus domestica	4	4	16512.81	3748.61	19858.41	11137.43
Malva moschata	5	3	1273.19	293.16	1523.88	783.99
Mercurialis perennis	5	3	9731.80	1206.43	11267.92	7884.40
Odontites vernus	3	3	14163.90	2713.54	16473.21	11175.23
Plantago coronopus	3	3	8646.77	1684.47	10471.89	7151.85
Plantago lanceolata	7	3	9205.73	1645.18	11387.39	6920.12
Primula vulgaris	8	3	7611.53	1654.30	10863.40	5842.85
Prunus avium	5	3	14347.66	5079.11	20662.50	9089.12
Prunus spinosa	5	4	12668.48	2747.69	14883.98	8061.50
Pulicaria dysenterica	4	4	2985.72	1141.45	4642.91	2208.35
Ranunculus acris	7	4	4619.54	659.70	5517.67	3768.29
Ranunculus bulbosus	6	3	4846.93	910.86	6033.13	4058.68

Ranunculus ficaria	8	4	5482.62	628.63	6584.11	4669.94
Ranunculus repens	4	3	4308.21	661.07	4985.55	3410.64
Reseda lutea	3	3	9977.46	2052.98	11566.78	7659.56
Rosa canina	4	3	6078.97	1957.45	8818.58	4485.81
Sanguisorba minor	4	3	12942.59	1427.02	14137.58	11224.99
Scorzoneroides autumnalis	3	3	15686.67	6467.89	23147.11	11656.49
Silene dioica	6	3	12283.42	804.68	13402.14	11328.09
Sonchus arvensis	3	3	38158.33	4914.28	41611.09	32532.08
Succisa pratensis	4	3	2616.96	208.29	2849.00	2434.76
Tripolium pannonicum	5	4	1308.60	618.60	1716.58	255.50
Ulex europaeus	5	3	7592.45	1999.13	9858.11	4941.57
Veronica chamaedrys	3	3	10931.14	1507.20	12666.23	9946.36

Table S6. Maximum, minimum, median and Inter Quartile Range (IQR) percentage of sterols which occurred at >40% in at least one species. Statistics were calculated from species/subspecies means, (genus where species was not available). Sterols which have a median of >2.5%, isofucosterol, avenasterol and β-sitosterol, are highlighted in grey. These sterols are positioned close together in the sterol biosynthesis pathway (Figure 2). For un-named sterols, a level two annotation (carbon count and double bond saturation) is shown in brackets.

Sterol	Max	Min	Median	IQR	Percentage of species detected in
24MC	91.03	0.00	1.51	11.47	99.3
Schottenol	88.24	0.00	0.25	5.04	99.3
Desmosterol	83.50	0.00	0.02	0.05	91.5
Cycloartenol	77.92	0.02	2.32	4.25	100.0
Isofucosterol	76.41	0.00	3.47	14.64	98.0
ST(29:1)C	70.94	0.00	0.13	0.33	98.0
Avenasterol	69.79	0.00	2.58	12.95	99.3
ST(30:2)B	65.65	0.02	0.22	0.48	100.0
β-Sitosterol	64.90	0.00	4.68	20.24	96.3
Cycloartanol	62.49	0.00	0.03	0.10	91.9
Episterol	61.76	0.00	0.02	0.14	69.8
Cholesterol	61.67	0.00	0.01	0.11	73.2
Cycloeucalenol	60.85	0.00	1.67	4.77	93.6
ST(27:2)D	60.06	0.00	0.00	0.02	79.0
ST(27:1)B	59.69	0.00	0.00	0.00	50.2
Obtusifoliol	56.75	0.00	1.11	3.05	88.1
Lathosterol	55.95	0.00	0.06	0.29	98.0
ST(30:1)	49.62	0.00	0.02	0.15	87.5
24MD	45.24	0.00	0.07	0.31	94.9
ST(30:2)A	44.07	0.00	0.19	0.24	99.7
ST(30:2)E	40.37	0.00	0.50	1.25	99.7

Table S7. Maximum, minimum, median and Inter Quartile Range (IQR) of sterols which were present in all taxonomic units analysed (minimum species value in dataset >0). Statistics were calculated from species means. For un-named sterols, carbon count and saturation are shown in brackets. Only four sterols out of 78 were detected in all taxonomic units. The two which could be named using reference materials do not share structural similarities and are many multiple metabolic steps apart in the sterol biosynthesis pathway (Fig 2).

Sterol	Max (%)	Min (%)	Median (%)	IQR (%)
Cycloartenol	77.92	0.02	2.32	4.25
ST(30:2)B	65.65	0.02	0.22	0.48
ST(30:2)C	36.51	0.04	0.74	1.15
Campesterol	30.23	0.01	1.78	3.90

\

Table S8. Highest and lowest Simpson's diversity index values across all pollen species. Highest values are shown in the left of the table and lowest values are in the right. These values demonstrate that even though pollen contains a wide range of sterols, they do not generally produce them in equal proportions.

Species	Simpson's diversity index	Species	Simpson's diversity index
Papaver cambricum	0.913	*Torminalis glaberrima*	0.169
Hypochaeris radicata	0.911	*Malus sp.*	0.204
Leontodon sp.	0.906	*Muscari armeniacum*	0.219
Campanula latifolia	0.904	*Hyacinthoides non-scripta*	0.262
Hedera helix	0.902	*Hyacinthoides x massartiana*	0.273
Anemonoides nemorosa	0.889	*Malus domestica*	0.273
Primula vulgaris	0.886	*Symphytum orientale*	0.295
Pulicaria dysenterica	0.886	*Rosa arvensis*	0.317
Geum urbanum	0.885	*Prunella vulgaris*	0.323
Phacelia tanacetifolia	0.884	*Crataegus monogyna*	0.347

Table S9. Sterols most commonly dominant in pollen, defined as occurring within the top three sterols by proportion. Sterols are ordered by number of taxonomic units for they were dominant in. Only two sterols were ranked in the top three for over 100 species; β-sitosterol and isofucosterol. Only 39/78 detected sterols ever occurred in the top three for any pollen species.

Sterol	No. of species ranked in top three by proportion	Sterol	No. of species ranked in top three by proportion
β-Sitosterol	127	ST(28:3)B	9
Isofucosterol	120	24MCA	8
Avenasterol	98	ST(28:1)A	8
24MC	89	ST(30:2)C	8
Schottenol	61	Cholesterol	7
Cycloeucalenol	55	ST(27:1)B	3
Campesterol	46	ST(27:2)E	3
Cycloartenol	44	ST(29:1)C	3
Obtusifoliol	38	Brassicasterol	2
ST(30:2)E	23	ST(27:1)D	2
Desmosterol	20	ST(30:1)	2
Cyclolaudenol	13	ST(31:2)A	2
ST(30:2)B	13	ST(31:2)B	2
Episterol	12	ST(28:3)A	1
24MD	11	ST(29:0)C	1
ST(30:2)F	11	ST(30:2)A	1
24MP	10	ST(30:2)D	1
Cycloartanol	10	Salisterol	1
Lathosterol	10	Stigmasterol	1
ST(27:2)D	9		

Table S10. Summary of Indicator Species Analysis (ISA) results comparing the sterol profiles of all three Asteraceae subfamilies sampled (Asteroideae, Cichorioideae and Carduoideae – main text Figure 3). Indicator values (IV) and p-values are shown for all significant associations. All three subfamilies were associated with at least six un-named sterols demonstrating how poorly characterised the Asteraceae currently sterolome is. Carduoideae was associated with the most sterols and showed the smallest variation in sterolome between species. Data were averaged by species and subfamilies contained different number of species; Asteroideae (21), Carduoideae (14) and Cichorioideae (17).

Sterol	IV	P-value	Sterol	IV	P-value
Asteroideae			Carduoideae		
Spinasterol	0.783	0.005	ST(27:2)F	0.847	0.005
ST(29:2)D	0.738	0.005	Lophenol	0.759	0.005
ST(29:3)B	0.704	0.010	ST(27:1)A	0.757	0.005
Sitostanol	0.690	0.025	ST(30:2)F	0.739	0.005
ST(28:0)E	0.684	0.005	Schottenol	0.724	0.005
ST(29:2)A	0.683	0.010	ST(29:3)A	0.713	0.030
ST(28:3)A	0.663	0.045	ST(29:0)C	0.708	0.005
Obtusifoliol	0.660	0.025	Cycloeucalenol	0.705	0.010
ST(28:3)B	0.655	0.035	ST(28:0)D	0.682	0.005
ST(28:0)A	0.556	0.040	ST(27:2)B	0.674	0.010
Cichorioideae			ST(31:2)A	0.673	0.005
ST(27:2)E	0.912	0.005	ST(28:2)B	0.670	0.015
Lathosterol	0.860	0.005	ST(30:2)E	0.668	0.050
Cycloartanol	0.823	0.005	ST(28:2)D	0.663	0.030
ST(30:2)B	0.815	0.005	ST(28:0)C	0.663	0.025
ST(27:2)G	0.767	0.025	ST(29:1)B	0.646	0.020
ST(29:1)C	0.762	0.005	ST(30:3)	0.643	0.045
ST(28:1)A	0.755	0.005	ST(30:2)A	0.637	0.005
ST(27:1)D	0.740	0.045			
24MC	0.707	0.005			
Brassicasterol	0.681	0.005			
Isofucosterol	0.660	0.005			

Table S11. Results of phylogenetic signal analysis on the proportions of all detected sterols with a maximum proportion >1% (69), total sterols (mg/kg), proportion of different carbon length sterols (light grey: 27, 28, 29, 30, 31) and proportion of different B-ring saturated sterols (dark grey: Cyclopropane ring (CPR), Δ0, Δ5, Δ7, Δ8, ΔNA). Only variables which were significant for both Bloomberg's K and Paegel's λ are shown. This includes 31/69 tested sterols and most structural groups, demonstrating the strong phylogenetic signal in the data. Ergosterol was omitted from this summary despite its significant phylogenetic signal as it is not part of the sterol synthesis pathway in plants.

Group	K	Lambda	p K	p λ	Group	K	Lambda	p K	p λ
24MC	0.202	0.951	0.001	0.001	ST(27:2)B	0.135	0.963	0.013	0.001
24MD	0.091	0.943	0.016	0.001	ST(28:2)B	0.363	0.818	0.003	0.001
24MP	0.050	0.881	0.035	0.001	ST(28:2)C	0.210	0.762	0.003	0.001
Agnosterol	0.071	0.615	0.015	0.001	ST(28:3)A	0.059	0.667	0.026	0.002
Anthelsterol	0.091	0.585	0.007	0.001	ST(28:3)B	0.979	0.817	0.001	0.001
Avenasterol	0.047	0.579	0.009	0.001	ST(29:0)C	0.061	0.951	0.002	0.001
β-Sitosterol	0.101	0.965	0.001	0.001	ST(29:1)A	0.259	0.998	0.012	0.001
Brassicasterol	0.227	1.000	0.004	0.001	ST(29:2)B	0.112	1.000	0.010	0.001
Campesterol	0.226	0.977	0.001	0.001	ST(30:2)E	0.080	0.952	0.015	0.001
Cycloartenol	0.129	0.986	0.013	0.001	ST(31:2)B	0.190	0.997	0.029	0.001
Desmosterol	0.091	0.950	0.015	0.001	Total sterol	0.070	0.814	0.010	0.001
Isofucosterol	0.078	0.834	0.006	0.001	27	0.094	0.962	0.003	0.001
Schottenol	0.047	0.898	0.034	0.001	28	0.252	0.980	0.001	0.001
Sitostanol	0.060	0.848	0.045	0.001	29	0.100	0.949	0.001	0.001
Spinasterol	0.144	0.990	0.007	0.001	30	0.067	0.919	0.001	0.001
Stigmasterol	0.083	0.987	0.013	0.001	CPR	0.059	0.919	0.007	0.001
ST(28:1)A	0.104	0.797	0.023	0.001	0	0.060	0.848	0.036	0.001
ST(27:1)A	0.386	0.997	0.007	0.001	5	0.069	0.879	0.001	0.001
ST(29:0)A	0.306	1.000	0.018	0.001	7	0.042	0.761	0.015	0.001
ST(29:2)D	0.132	0.961	0.008	0.001	NA	0.033	0.724	0.047	0.001

Table S12. Summary of Indicator Species Analysis (ISA) results on pollen sterol data comparing genera within families (Figure 4A). Out of 78 sterols tested, Asteraceae genera showed associations with 42, Fabaceae with 26 and Rosaceae with 38. Indicator values (IV) and p-values are shown for all. Data were analysed separately by family, each genus contained at least three species.

Sterol	IV	P-value	Sterol	IV	P-value	Sterol	IV	P-value
Asteraceae			Fabaceae			Rosaceae		
Centaurea			*Trifolium*			*Potentilla*		
Cyclolaudenol	0.804	0.005	Schottenol	0.856	0.005	ST(27:2)D	0.987	0.005
Sitostanol	0.801	0.005	ST(27:2)C	0.785	0.005	ST(29:0)A	0.952	0.005
ST(28:0)E	0.792	0.005	24MC	0.754	0.005	Schottenol	0.940	0.005
ST(28:2)B	0.772	0.005	Ergosterol	0.670	0.015	ST(28:3)A	0.934	0.005
ST(27:2)B	0.736	0.005	Spinasterol	0.628	0.005	24MP	0.928	0.005
ST(29:2)A	0.736	0.005	*Ulex*			Brassicasterol	0.875	0.020
ST(30:3)	0.734	0.005	Sitostanol	0.891	0.005	ST(27:2)E	0.849	0.015
ST(27:1)E	0.724	0.010	ST(28:0)E	0.888	0.005	ST(27:1)C	0.843	0.005
ST(31:2)A	0.701	0.005	ST(30:2)E	0.771	0.005	Lathosterol	0.814	0.005
Ergosterol	0.686	0.010	ST(30:1)	0.748	0.015	Episterol	0.789	0.025
24MCA	0.674	0.015	Stigmasterol	0.748	0.020	Spinasterol	0.766	0.005
24MD	0.655	0.040	ST(29:1)D	0.745	0.010	ST(29:3)A	0.765	0.005
ST(30:1)	0.650	0.030	Cycloeucalenol	0.742	0.005	ST(29:2)C	0.751	0.025
ST(27:2)C	0.636	0.035	β-Sitosterol	0.725	0.020	ST(29:1)B	0.744	0.005
Cirsium			ST(30:2)B	0.677	0.010	Campesterol	0.716	0.005
ST(27:1)B	0.916	0.005	ST(29:0)C	0.673	0.010	ST(29:2)B	0.704	0.045
ST(27:2)F	0.898	0.005	ST(30:2)A	0.664	0.005	ST(29:1)A	0.701	0.020
ST(27:1)A	0.810	0.005	Campesterol	0.652	0.035	Cycloartenol	0.692	0.020
ST(29:0)B	0.757	0.005	ST(30:2)C	0.641	0.010	ST(29:0)C	0.675	0.040
ST(30:2)E	0.713	0.010	*Vicia*			*Prunus*		
Cycloeucalenol	0.700	0.005	ST(29:1)C	0.755	0.040	ST(28:0)A	0.840	0.010
Spinasterol	0.695	0.005	ST(29:3)A	0.737	0.005	ST(31:2)A	0.804	0.005
ST(27:2)A	0.694	0.005	24MCA	0.729	0.015	ST(28:2)B	0.796	0.005
Schottenol	0.689	0.005	Anthelsterol*	0.716	0.005	Cyclolaudenol	0.791	0.005
Lophenol	0.686	0.010	Cyclolaudenol	0.696	0.005	ST(28:3)B	0.771	0.025
24MP	0.677	0.015	ST(31:2)A	0.664	0.005	Isofucosterol	0.758	0.005
ST(29:0)C	0.677	0.005	Cycloartenol	0.620	0.030	24MC	0.741	0.015
ST(29:1)A	0.664	0.045	ST(28:0)B	0.577	0.020	β-Sitosterol	0.731	0.005
ST(30:2)B	0.659	0.005				ST(28:2)C	0.708	0.025
Avenasterol	0.656	0.005				ST(28:1)A	0.707	0.045
ST(30:2)C	0.646	0.005				Obtusifoliol	0.691	0.040
Crepis						*Rubus*		
ST(28:3)B	1.000	0.005				Desmosterol	0.992	0.005
ST(27:2)E	0.889	0.010				ST(30:2)D	0.918	0.005
ST(28:1)A	0.857	0.005				Stigmasterol	0.810	0.010
ST(29:1)C	0.846	0.010				24MCA	0.804	0.010
Cycloartanol	0.833	0.005				ST(30:2)E	0.757	0.005

24MC	0.791	0.005
ST(29:0)A	0.777	0.010
Brassicasterol	0.768	0.005
Obtusifoliol	0.708	0.010
Isofucosterol	0.707	0.005
Lathosterol	0.706	0.005
Cycloartenol	0.674	0.020

ST(27:2)A	0.756	0.005
ST(27:1)E	0.727	0.015
ST(30:2)C	0.666	0.005

Table S13. Summary of Indicator Species Analysis (ISA) results on pollen sterol data for the inter-species comparison shown in Figure 4B,C. Out of 78 sterols tested, 45 were significantly associated with a species group. Indicator values (IV) and p-values are shown for all 45. All species are associated with at least three sterols, indicating they have reliably distinct sterolomes as shown in Figure 4B,C. *Prunus spinosa* demonstrated the greatest variance in sterol profile and has the weakest sterol associations.

Sterol	IV	P-value	Sterol	IV	P-value
Calluna vulgaris			*Ranunculus ficaria*		
Sitostanol	0.805	0.005	ST(29:2)B	0.734	0.005
ST(30:2)F	0.638	0.040	Isofucosterol	0.627	0.005
β-Sitosterol	0.625	0.005	ST(31:2)B	0.580	0.045
Stigmasterol	0.592	0.005	ST(30:2)A	0.530	0.005
ST(29:2)A	0.555	0.005	Obtusifoliol	0.522	0.005
ST(29:1)B	0.545	0.005	*Tripolium pannonicum*		
Hyacinthoides non-scripta			ST(27:1)B	0.991	0.005
ST(29:3)A	0.785	0.005	ST(28:0)C	0.758	0.010
Schottenol	0.773	0.005	ST(27:1)E	0.736	0.005
ST(28:2)A	0.703	0.005	ST(30:1)	0.687	0.005
ST(28:1)A	0.694	0.005	ST(30:2)D	0.668	0.010
ST(29:2)D	0.657	0.030	ST(29:0)A	0.643	0.005
Cycloartanol	0.607	0.005	Spinasterol	0.622	0.005
Knautia arvensis			ST(27:2)B	0.612	0.005
24MP	0.644	0.005	Avenasterol	0.556	0.005
ST(30:2)E	0.599	0.010	Cycloeucalenol	0.555	0.010
ST(27:1)C	0.585	0.025	ST(28:2)B	0.552	0.005
Prunus spinosa			ST(27:2)A	0.549	0.005
ST(28:3)B	0.586	0.020	ST(28:0)E	0.534	0.005
24MC	0.578	0.020	ST(30:2)C	0.474	0.005
Cyclolaudenol	0.498	0.005			
Ranunculus acris					
Ergosterol	0.826	0.005			
ST(28:3)A	0.658	0.010			
ST(27:2)E	0.627	0.005			
Cycloartenol	0.576	0.005			
Campesterol	0.572	0.005			
Lathosterol	0.572	0.010			
24MD	0.518	0.010			
ST(29:1)A	0.503	0.005			

References

Zanne, A. E. *et al.* (2014) ‘Three keys to the radiation of angiosperms into freezing environments’, *Nature*, 506, pp. 89–92. doi: 10.1038/nature12872.
